# Editorial: Plant-Microbe-Insect Interaction: Source for Bio-fertilizers, Bio-medicines and Agent Research

**DOI:** 10.3389/fpls.2018.00931

**Published:** 2018-06-28

**Authors:** Gero Benckiser, Anton Hartmann, Krishnamurthy Kumar, Bernd Honermeier

**Affiliations:** ^1^Applied Microbiology, Justus Liebig Universität Gießen, Giessen, Germany; ^2^Deutsches Forschungszentrum für Gesundheit und Umwelt, Helmholtz Zentrum München, Munich, Germany; ^3^Department of Agricultural Microbiology, Tamil Nadu Agricultural University, Coimbatore, India; ^4^Department of Agronomy and Plant Breeding, Justus Liebig Universität Gießen, Giessen, Germany

**Keywords:** tripartite sebacinalean symbiosis, MATI regulation, virulence reduction, antimicrobial activity, nematode subpressing

The plethora of taxonomically, phylogenetically, and metabolically diverse soil- microbes, plants and animals is responsible for the dynamic observed in ecosystems. Especially the above- and below-ground species flexibility that drives biogeochemical processes is not well understood but is of great significance for food production networks and yield stability (Benckiser, 1997; Benckiser and Schnell, 2007)

Ecosystem management and agricultural praxis have until now been largely conducted in isolation but the recent interconnection of inextricably various disciplines has now given rise to a more holistic approach. This Frontiers' e-book on “Plant-Microbe-Insect Interaction: Source for Bio-fertilizers, Bio-medicines and Agent for Research” presents 42 contributions covering a broad spectrum of modern physico-chemical, biochemical, molecular biology and agronomy techniques with the aim to provide perspectives on how a better productivity, health and fitness could be achieved. The focus lies in the long term adapted composition and functioning of microbe-insect interactions, which should be brought in line with agricultural productivities.

In modeling of biological networks and system biology approaches Kumar et al. are introducing and giving insights into Indian rice landrace diversity Imam et al. as well as Saxena et al. and Wei et al. on anthracnose management in chilli and wide range of cruciferous crops. The cofactor vitamin B1 plays an important role in metabolic reactions of all living organisms and it is not surprising that thiamine biosynthesis of endophytic *Henderosonia toruloidea* colonizing palm oil seedlings improves their growth (Kamarudin et al.). In reports on two- or tripartite mutualistic symbioses Hashem et al. address the symbiosis between arbuscular mycorrhizal fungi (AMF) and “Formosa” azalea, *Azalea gerrardii*, Wei et al. describe how the mycorrhizal fungus *Oidiodendron maius* positively influences the growth of *Rhododendron fortunei* Lindl., Guo et al. describe fitness rise in a cooperation of *Rhizobium radiobacter, Piriformospora* and cereal crops, and Sharma et al. found salt stress adaptation of peanuts in the presence of halotolerant, endophytic, plant growth promoting rhizobacteria (PGPR). The endophyte *Acidovorax radicis* N35, a major producer of *N*-acyl homoserine lactone (AHL) type, favors growth of barley (Han et al.), the medicinal plant *Hypericum perforatum* shows a remarkable activity against bacterial and fungal pathogens (Egamberdieva et al.), and bacteria identified by pyrosequencing combined with qPCR effectively suppress *Fusarium* wilt in the Chinese medicinal plant *Pseudostellariae heterophylla* (Wu et al.).

A selective pressure niche to plant growth promoting bacteria (PGPB) evolution is the Brazilian Iron Quadrangle (IQ) soil (Felestrino et al.). IQ soil is a specific reservoir for PGPB species useable in reforestation of antropized soil and one of those could be *Pseudomonas aeruginosa* PM12, which induces systemic resistance in tomato against Fusarium wilt (Fatima and Anjum). Phenolic, allelochemically acting acids apparently mediate shifts in soil rhizosphere microbial flora, which inter alia ameliorate tomato Fusarium wilt resistance in mono-cropping systems of the medicinal plant *Radix pseudostellariae* (Wu et al.).

The TIFY domain of poplar species contains approximately 36 conserved amino acids (Xia et al.). The forming core motif TIF[F/Y]XG plays a role in poplar growth, tissue development, and defense regulation. Fungus-like growing rhizosphere actinomycetes are known to promote growth and yield enhancement in wheat crops through the plant growth hormone indole acetic acid (IAA) and among rhizospheric IAA producers the most active and good candidates for developing biofertilizers are *Streptomyces nobilis, Streptomyces kunmingenesis*, and *Streptomyces enissocaesilis* (Anwar et al.). A candidate for agro-active compound development is also *Streptomyces hydrogenans*, (Manhas and Kaur). This non-cytotoxic, non-mutagenic, 10-(2,2-dimethyl-cyclohexyl)-6,9-dihydroxy-4,9-dimethyl-dec-2-enoic acid methyl ester producing actinomycete causes severe morphological alterations in *Alternaria brassicicola* (Kaur et al.).

Besides *Streptomyces* species, *Bacillus cereus* AR156 is also a suppressing agent (Nie et al.). AR156-treated *Arabidopsis* plants activate MAPK signaling and *FRK1*/*WRKY53* gene expression, both of which are involved in pathogen associated molecular pattern (PAMP)-triggered immunity (PTI) and suppress *Pseudomonas syringae* pv. *tomato* DC3000 (*Pst* DC3000). The strength of induced systemic resistance (ISR) against *Botrytis cinerea* due to these bacteria remains unknown (Nie et al.). A common bacterial rice pathogen is the virulent, biofilm forming *Xanthomonas oryzae* pv. *oryzae* (Singh et al.). With a combined application of Thyme oil (THY) and conventional bactericides the *motA, motB, flgE, rpfF* gene activity can be reduced, extracellular endoglucanase, xylanase, cellobiosidase, and polygalacturonase genes are expressed, the bactericide dose can be lowered, and disease progression be diminished.

An antioxidative amino acid is the histidine-derived, sulfur-containing, non-proteinogenic ergothioneine (EGT) that adds to phyllospheric fitness (Alamgir et al.). *M. aquaticum* strain 22A produces high amounts of EGT. It has been isolated from a moss and has been only poorly studied. This is also the case for the facultative methylotrophic, pink pigmented bacteria (PPFM), identified as *Delftia lacustris, Bacillus subtilis*, or *B. cereus* by 16S rRNA gene sequence analysis. They are directly antagonistic against fungal pathogens of tomato and regarded as biocontrol agents (Janahiraman et al.). Out of 217 rhizobacterial isolates obtained from six different Indian Assam tea estates, Dutta et al. identified *Enterobacter lignolyticus, Bacillus pseudomycoides, Burkholderia* sp., or *P. aeruginosa* by partial 16S rRNA gene sequence analysis and as bio-fertilizer candidates for tea crops. Tamilselvi et al. identified *Brevibacterium* sp. SOTI06 as calcite dissolving bacterium, Krithika and Balachandar identified *Bacillus* sp. or *Enterobacter cloacae* as zinc (Zn) solubilizing bacteria, and Shakeel et al. reports that *E. cloacae* releases under iron- sufficient and -deficient conditions Zn-regulated transporters and iron (Fe)-regulated transporter-like proteins (ZIP) into the rice rhizosphere. Islam et al. assessed by quantitative real-time reverse transcription PCR that the 234 isolates obtained from the roots of basmati-385 and basmati super rice varieties translocate Zn toward grains and suppress economically important rice pathogens as *Pyricularia oryzae* and *Fusarium moniliforme*. In Bangladesh, *Pseudomonas stutzeri, B. subtilis, Stenotrophomonas maltophilia, Bacillus amyloliquefaciens*, present in cucumber rhizosphere and identified by phylogenetic 16S rRNA sequences, characteristically change the morphology of *Phytophthora capsici* hyphae growing toward PGPR colonies and from *B. amyloliquefaciens* (SN13) it is known that it prolongs stress tolerance in rice and acts as biocontrol agent against *Rhizoctonia solani* (Srivastava et al.). The virulence of fruit rot fungi during cranberry fruit development influences the release of ROS suppressive compounds such as benzoic and quinic acids (Tadych et al.) and intercropping can minimize the success of pea weevil larvae (*Bruchus pisorum* L.; Teshome et al.). Some genotypes have the capacity to obstruct pea weevil larval entry into developing seeds [neoplastic (*Np*) genotypes] of *P*. *sativum* and intercropping *Np* genotypes with other crops such as sorghum and maize can facilitate neoplasm formation. Insect-plant-bacteria interaction is also the subject of Harun-Or-Rashid et al., who studied the widespread yield loss of many crops caused by the green peach aphid, a most destructive, plant sap sucking and plant viruses transmitting insect pest. Harun-Or-Rashid et al. found that *Bacillus velezensis* YC7010 which induces systemic resistance against bacterial and fungal pathogens of rice, can also induce systemic resistance against *the* green peach aphid (GPA), *Myzus persicae*, puncturing *Arabidopsis* leaves. *B. velezensis* significantly reduces setting, feeding and reproduction of GPA on *Arabidopsis* leaves by strongly inducing the senescence-promoting gene *PHYTOALEXIN DEFICIENT4* (*PAD4*) while suppressing *BOTRYTIS-INDUCED KINASE1* (*BIK1*). Another important soil-borne pathogen that affects field crops worldwide is *Heterodera avenae. Trichoderma longibrachiatum* has the ability to control *H. avenae* by surrounding the eggs completely with dense mycelia, lysing the content at late egg stage, and inducing a defense response in wheat plants (Zhang et al.). Insect-plant-bacteria interactions are also observable in citrus plants (Prasad et al.; Lin et al.). The destructive citrus disease, Huanglongbing, breaks out because in sap-sucking psyllids (*Diaphorina citri*) and *Candidatus* (*Ca*.) *Liberibacter asiaticus* infected citrus plants, the volatile cues are modified in a way that the *D. citri* predator, ladybird beetle *P. japonica* cannot find the host plant and reduce the insect pathogen, *D. citri*, population.

Plant health and productivity is of complex nature and strongly influenced by the intimate interaction of plants with deleterious and beneficial organisms. Obligate parasites affecting plant phloem such as *D. citri* and *Candidatus* (*Ca*.) *Liberibacter* link cellular levels with interdisciplinary scales and in such systems also viruses play a key role as the tomato—whitefly—Tomato Yellow Leaf Curl Virus (TYLCV) system studied by Tan et al.. Viral influence is also asserted by Li et al., who report that soil bensulfuron-methyl residues exhibit significant effects on the infestation of *Bemisia tabaci, Myzus persicae*, and *Tobacco mosaic virus* in *Nicotiana tabacum* and Trivedi et al. expanded such insights by highlighting the molecular, ecological, and evolutionary aspects of interactions among insects, plants, and their associated microbial communities. Islam et al., having nitrogen in their research focus, detail that a high level of nitrogen makes tomato plants release less volatiles and attract more *Bemisia tabaci* (Hemiptera: Aleyrodidae) and Santamaria et al. highlight that MATI proteins unveil a potential in defense through photosynthetic pigment modulation.

The *Achnatherum sibiricum* article of Qin et al. adds an aspect to the “Frontiers research topic Plant-Microbe-Insect Interaction: Source for Bio-fertilizers, Bio-medicines and Agent Research”, worthwhile to have a closer look. They report in their e-book contribution that *A. sibiricum*, a little-studied, native grass of the Inner Mongolian steppe, is highly infected by endophytes but is not producing detectable endophyte-related alkaloids as known from agronomically important grasses such as tall fescue and perennial ryegrass. Despite the herbivore resistance, the resistance to hosting *Locusta migratoria* increases by mechanisms different from endophyte-conferred and alkaloid decline.

All the articles contributed to the research topic “Plant-Microbe-Insect Interaction: Source for Bio-fertilizers, Bio-medicines and Agent Research” reveal interesting interactive details, and show that our understanding of the complexity of agricultural production systems is only in its very beginning. Nevertheless, the given insights in soil properties-virus-microbe-plant-insect interactions are hopefully a stimulus for continuing the discussion in this field.

## Author contributions

All authors listed have made a substantial, direct and intellectual contribution to the work, and approved it for publication.

### Conflict of interest statement

The authors declare that the research was conducted in the absence of any commercial or financial relationships that could be construed as a potential conflict of interest.
